# Conservative Management of Complicated Crown-Root Fracture: An Immediate Esthetic Rehabilitation

**DOI:** 10.7759/cureus.25627

**Published:** 2022-06-03

**Authors:** Rutuja Rajnekar, Nikhil Mankar, Pradnya Nikhade, Manoj Chandak, Karuna Burde

**Affiliations:** 1 Conservative Dentistry and Endodontics, Sharad Pawar Dental College and Hospital, Datta Meghe Institute of Medical Sciences (Deemed to be University), Wardha, IND; 2 Public Health Dentistry, Saraswati-Dhanwantari Dental College & Hospital, Parbhani, IND

**Keywords:** complicated crown-root fracture, endodontic therapy, reattachment, tooth fracture, dental trauma

## Abstract

Among the various types of dental trauma, crown-root fractures are one of the most challenging to treat and require a multidisciplinary approach. This paper reports a case of a complicated crown-root fracture of maxillary left central incisor with esthetic, functional complications. An 18-year-old male patient presented to the department immediately after suffering trauma with a complicated crown-root fracture on tooth 21. As per the treatment, the patient had undergone endodontic therapy followed by flap reflection. Post flap reflection, a glass fiber post was luted, and the fractured fragment was reattached. By this approach, in the same appointment, the cervical margin can be exposed with appropriate isolation followed by a reattachment procedure. Reattaching the fragment is a viable option as it can be done immediately, provides better esthetics, restores function, and is less complicated than the conventional approach. A good prognosis is dependent on patient cooperation with the understanding of the treatment limitations. The article discusses a successful case of complicated crown-root fracture treated with the reattachment of a tooth fragment.

Eighteen months of clinical and radiographic evaluation revealed that the clinical protocol was effective, as the tooth was functional, asymptomatic, and esthetic.

## Introduction

An increased incidence of traumatic dental injuries occurs as a result of recreational activities such as sports, where the crown fracture is the most common. This can range from simple enamel-dentin fractures to complicated crown-root fractures or root fractures [1]. A total of 25% of the population under the age of 18 years is estimated to suffer an anterior tooth fracture due to traumatic injury [2]. Of which, 80% are central incisors, whereas 16% are lateral incisors, as a result of their position and protrusion, which is due to the eruption process. Most traumatic injuries involve enamel and dentin fractures, whereas crown and root fractures that expose pulp constitute only 5-8% of all fractures. Based on a published case series, 85% of the fractures run obliquely from the buccal to the lingual, with the fracture line progressing apically [3]. The type and location of fracture vary depending on the patient's age, the amount of force applied, and the direction of the blow [4]. To restore the fractured tooth, different methods and techniques are recommended. In the late 1960s, the temporary, as well as permanent, restorations of traumatized teeth in young patients were difficult. Various methods of restoration such as resin crowns, stainless steel crowns, inlays with pin-retention, and complex ceramic restorations were used [5]. In addition to jeopardizing the tooth structure, these techniques were also esthetically unacceptable. Furthermore, in case of esthetic emergencies, these methods cannot be utilized [6]. In the 1970s, the adhesive composite restorations became almost a gold standard for the crown fracture treatment in children, adults, and at times even in older individuals. Additional treatment alternatives that are available as well are porcelain laminate veneers, porcelain fused to metal crowns, and all-ceramic crowns. The management of complicated crown-root fracture in young patients is challenging as the fracture line is below the bone crest and the pulp is exposed. Various treatment options include crown lengthening, orthodontic extrusion, and intentional replantation. In recent years, advancing technologies in acid-etching techniques and dentinal adhesives have led to an increase in minimally invasive approaches among dentists, so as in tooth reattachment procedures [7]. This can be achieved by preserving and retrieving traumatized tooth fragments. In comparison with conventional composite restoration, tooth fragment reattachment offers conservatism, a favorable wear mechanism, color matching with the remaining crown portion, preservation of incisal translucency, preservation of the same occlusal contacts and natural tooth contours, the color stability of the enamel, as well as ease of treatment and cost-effectiveness [8]. Reattachment serves as an interim restoration for young individuals who may need definitive procedures like direct adhesive veneer or crown if it fails. This article describes the successful treatment of fractured maxillary central incisor by reattachment procedure.

## Case presentation

**Figure 1:**
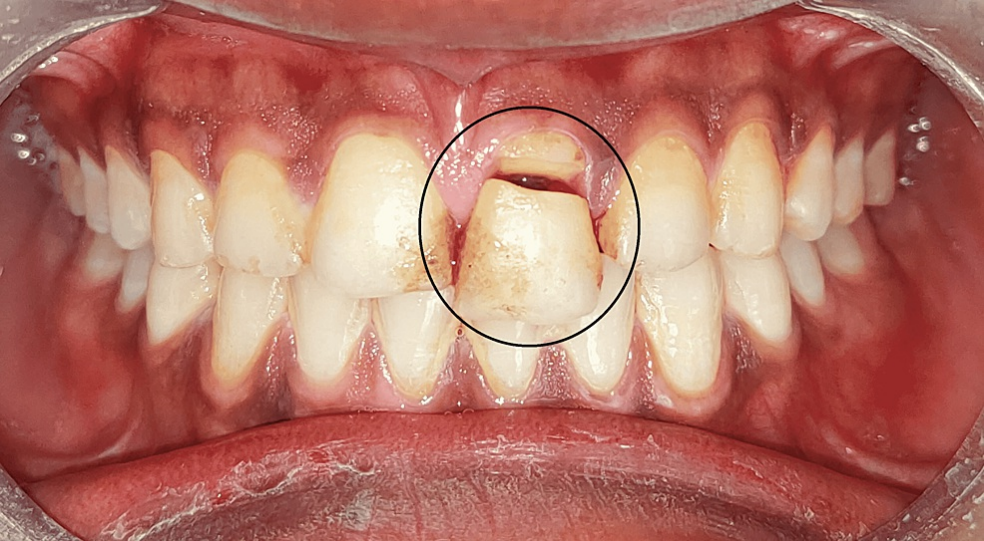
Preoperative clinical image of tooth 21

An 18-year-old male came to the department with a chief complaint of a fractured tooth in the upper front region of the jaw for two days. He reported that he had a sports injury in the upper front region of the jaw and experienced a fracture in the upper tooth. The fractured fragment was mobile and was associated with intermittent pain. Medical and dental history was not relevant. An extraoral clinical examination revealed well-coordinated temporomandibular joint (TMJ) movements. The intraoral examination revealed an oblique fracture with tooth 21 extending subgingivally. The fracture margin on the palatal surface was approximately 1.5 mm from free gingival margins. Fragment or part of the tooth was grade III mobile but was supported and intact palatally. Gingival tissue secured the fragment of the tooth (Figure 1).

**Figure 2:**
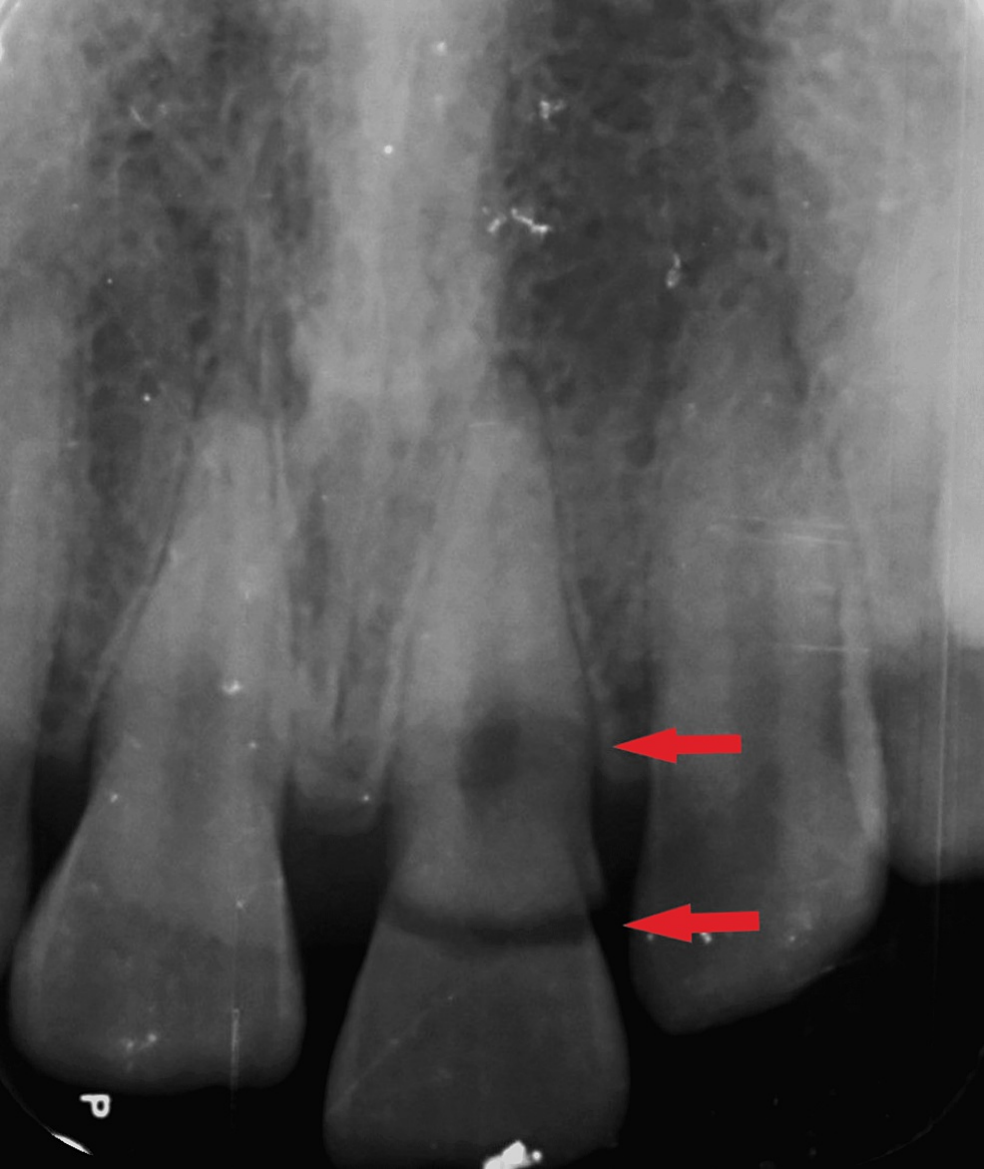
Preoperative radiograph of tooth 21 Preoperative radiograph showing fracture line running obliquely from the buccal to the palatal aspect of tooth 21.

On radiographic examination, there was an oblique fracture line going subgingivally with associated pulp exposure running labially to the palatal in an apical direction. No periradicular changes were observed (Figure 2).

The diagnosis was irreversible pulpitis with tooth 21 exhibiting oblique complicated crown-root fracture. Several treatment options were explained to the patient, with their pros and cons, the associated cost, and the prognosis. The decision for reattachment was made only after inspecting the fragment condition and its fit on the fractured tooth. A single-visit root canal treatment was planned for tooth 21, followed by reattachment of fragment by fiber post reinforcement.

**Figure 3:**
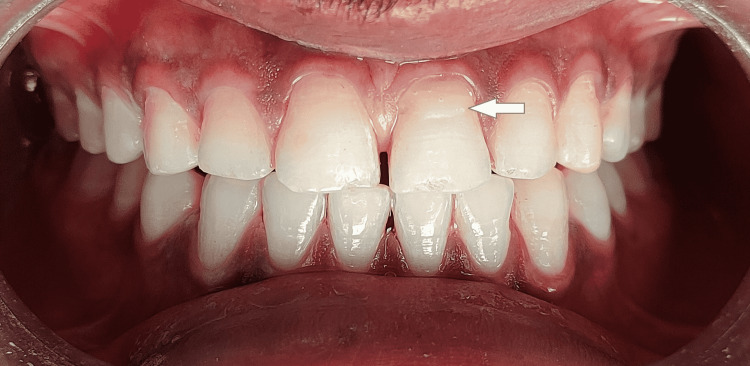
Temporary reattachment of the fractured fragment of tooth 21 by flowable composite

Under local anesthesia, temporary reattachment of the fractured fragment was done by flowable composite on the buccal aspect of tooth 21 (Figure 3).

**Figure 4:**
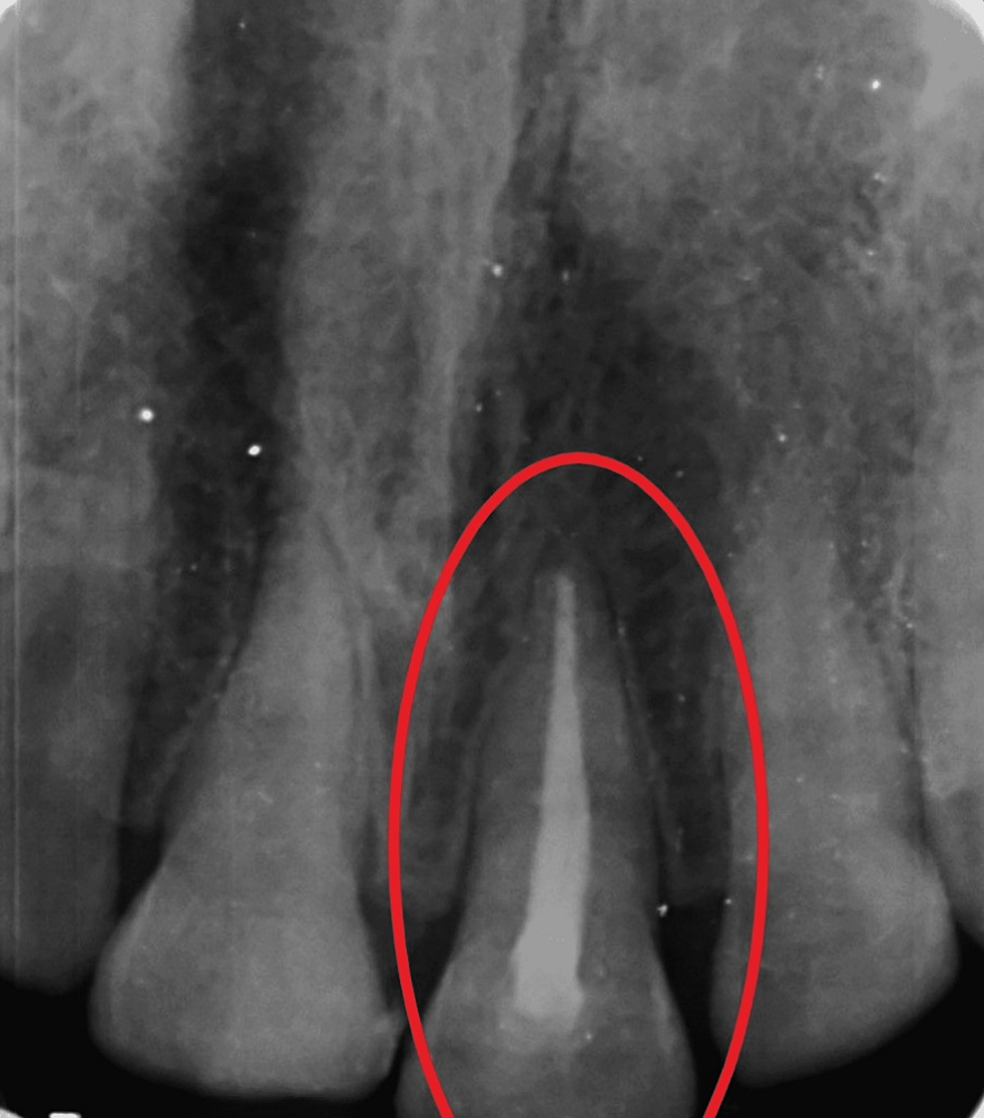
Complete root canal treatment of tooth 21 After completion of single-visit root canal treatment of tooth 21.

Access opening was prepared with minimal tooth structure removal, and a complete root canal was performed (Figure 4).

**Figure 5:**
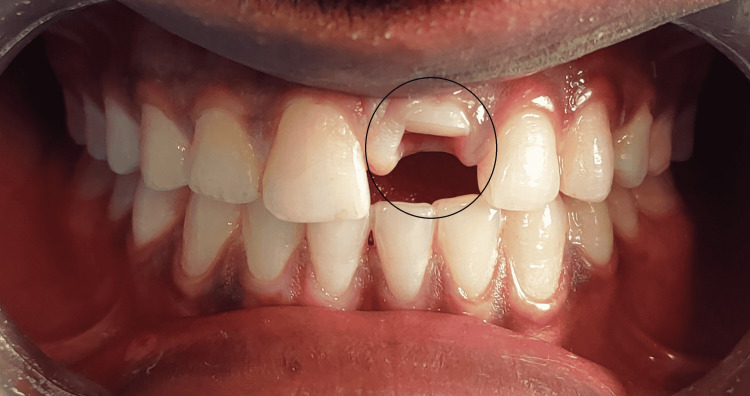
Clinical picture after removal of the fractured fragment from tooth 21

**Figure 6:**
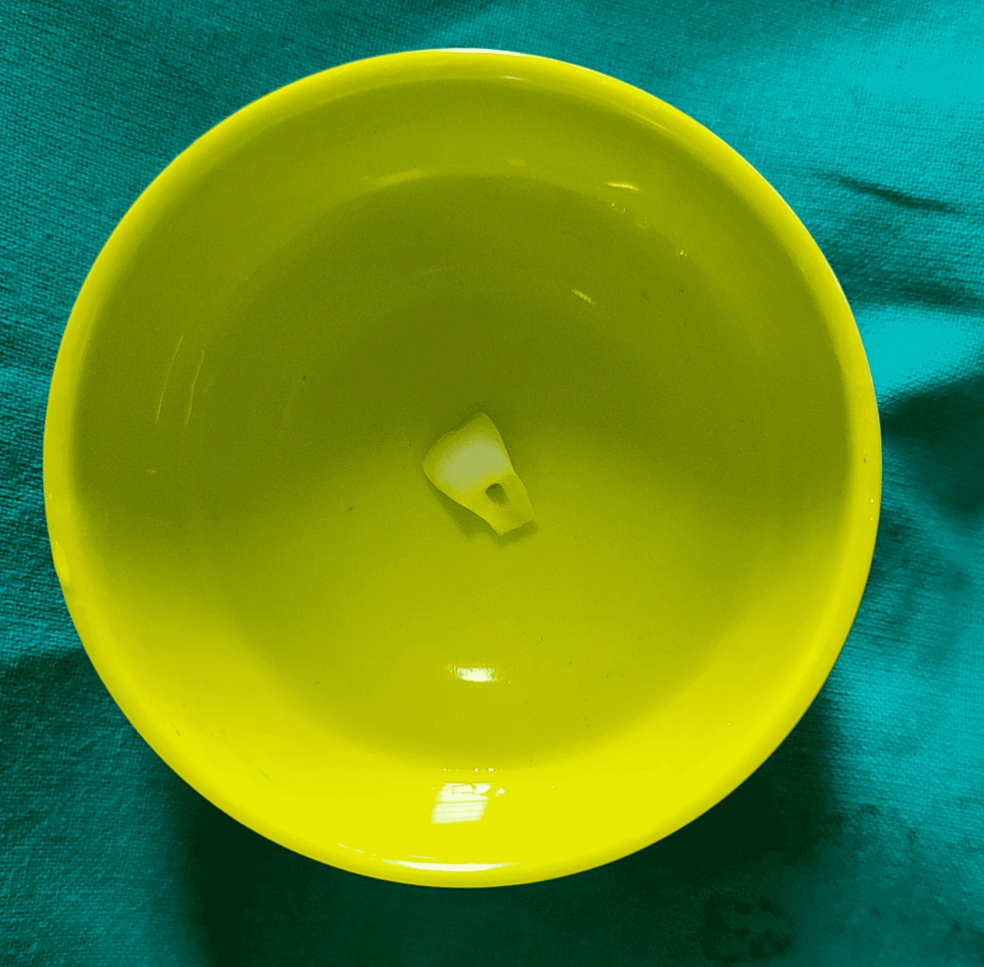
After removal of the fractured fragment, the segment was kept in normal saline to prevent dehydration till the reattachment

The fractured segment was separated (Figure 5) and kept in normal saline (Figure 6).

**Figure 7:**
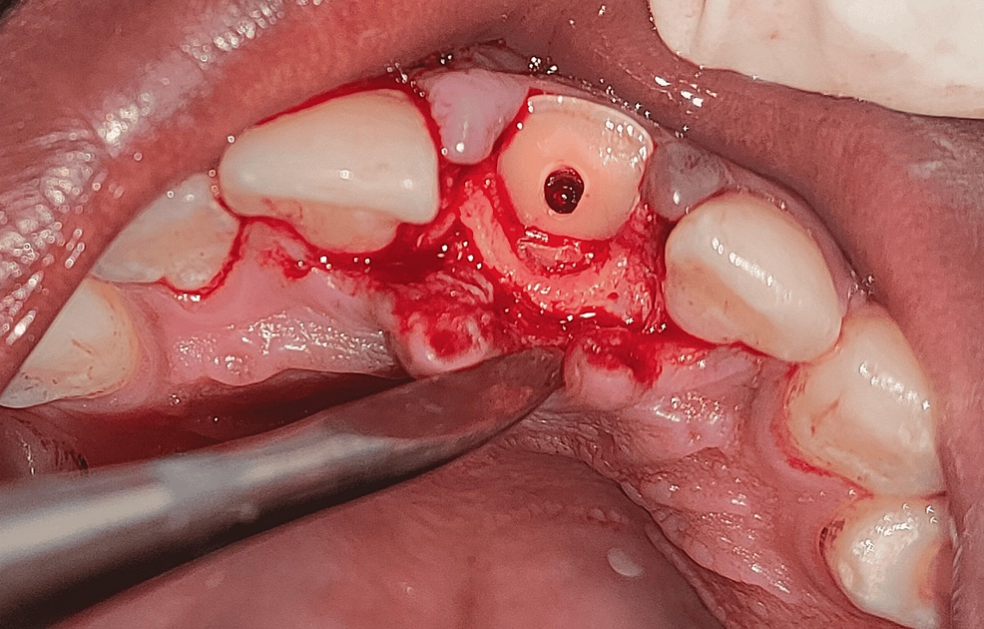
Periodontal flap reflection using the periosteal elevator in relation to tooth 21 Image showing raised palatal and buccal envelope flap by giving crevicular incision from # 11 to # 22 and reflecting using a periosteal elevator.

A palatal flap was raised to ensure proper attachment of the fragment, as it was fractured subgingivally from the palatal aspect. After local anesthesia, the palatal and buccal envelope flap was raised by giving a crevicular incision from distal of 11 to distal of 22 and reflecting using a periosteal elevator (Figure 7).

**Figure 8:**
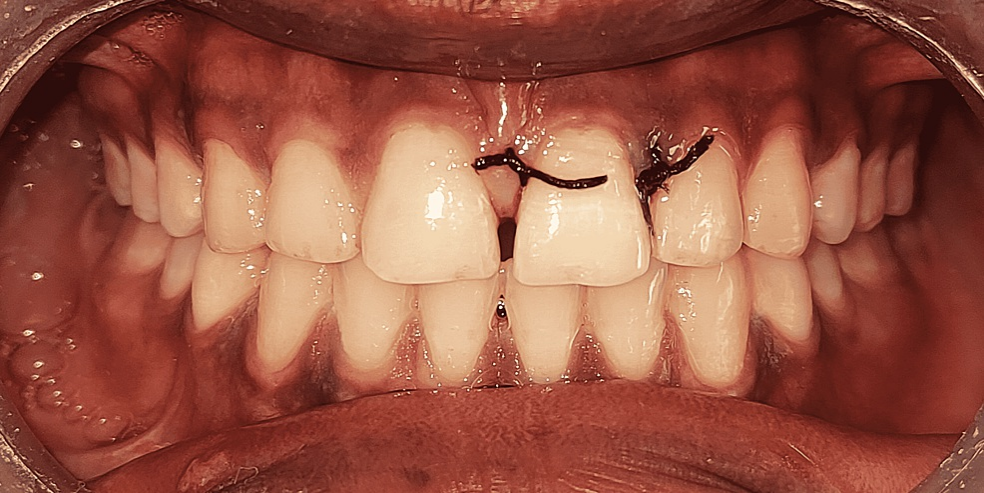
Immediate postoperative clinical picture after fractured fragment reattachment with tooth 21

**Figure 9:**
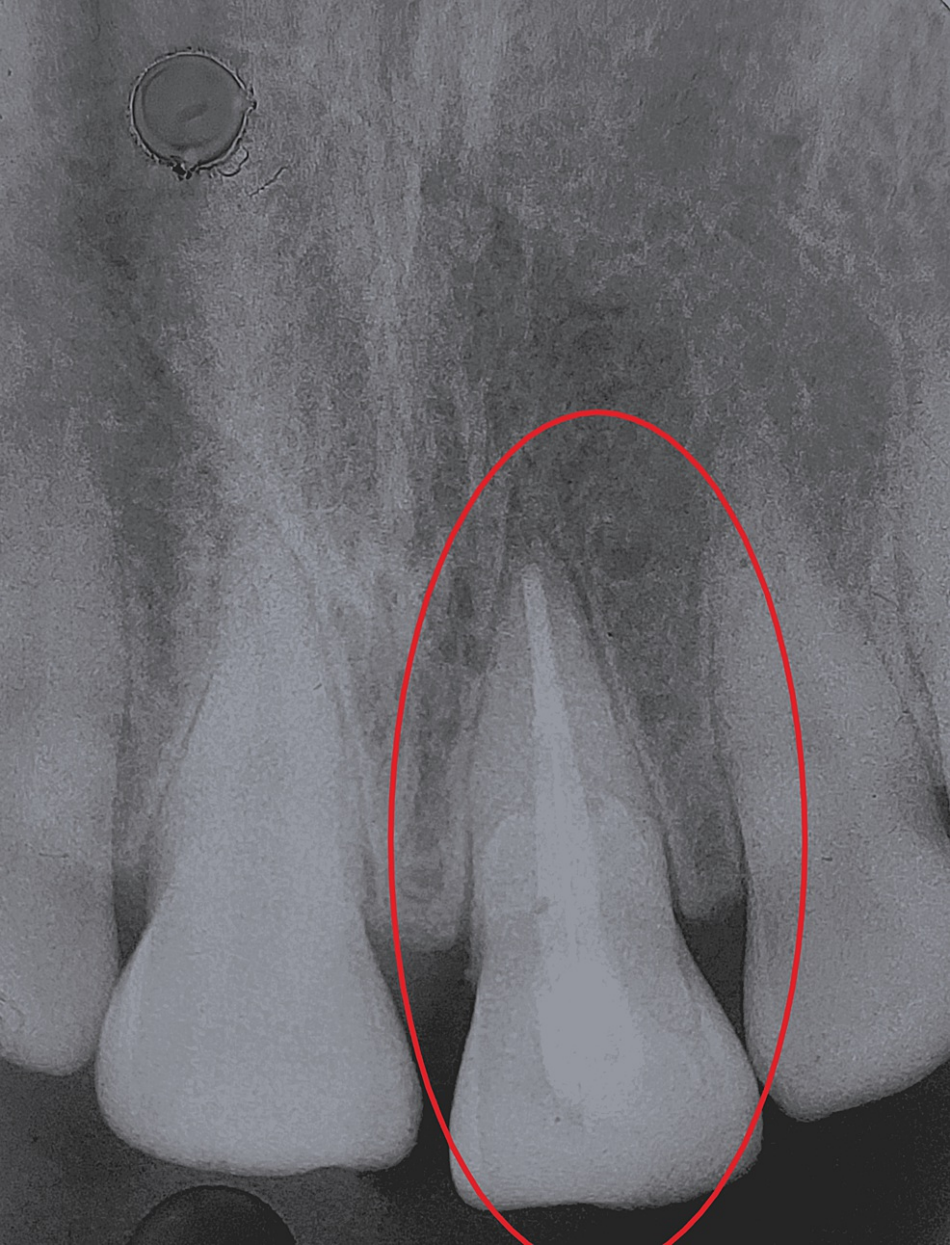
Immediate postoperative radiograph after fractured fragment reattachment with tooth 21

The canal was etched (etching gel, Prime Dental, Maharashtra, India) for 20 seconds, followed by rinsing with water and air-dried. The Prime & Bond NT adhesive and self-cure activator (Dentsply, Charlotte, North Carolina) were mixed and applied, and the teeth were light-cured for 20 seconds. The same applied to the post. The canal was injected with dual-cure resin (Calibra, Dentsply) followed by insertion of glass fiber post (size 1, Reforpost, Angelus, Londrina, Brazil) and final light curing for 20 seconds. The fractured fragment was reattached to the remaining tooth structure by using dual-cure resin (Calibra, Dentsply). The restorative margins were finished by using diamond burs and Sof-Lex Discs (3M ESPE, Seefeld, Germany), and diamond polishing paste was used for polishing (Figures 8, 9). The patient was instructed not to apply heavy pressure to these teeth and to follow proper oral hygiene practices.

**Figure 10:**
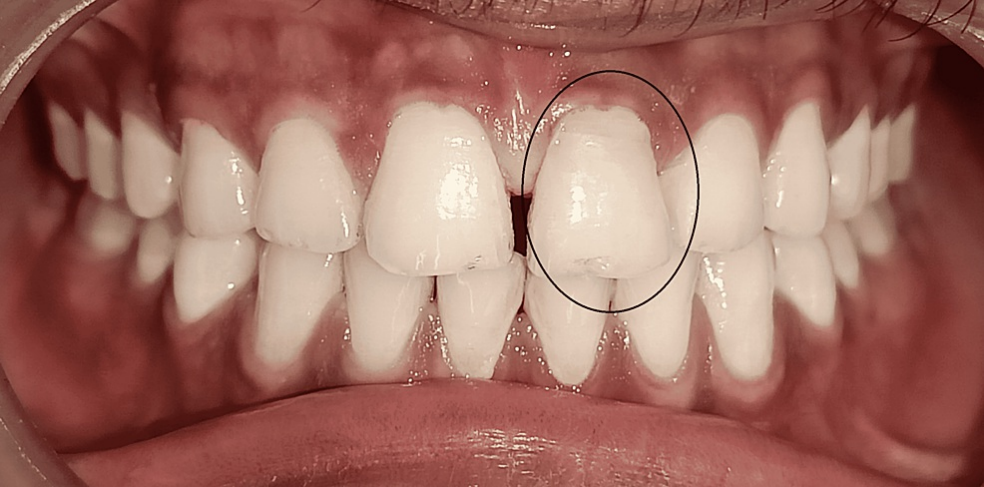
Clinical evaluation of tooth 21 after 18 months of fragment reattachment

**Figure 11:**
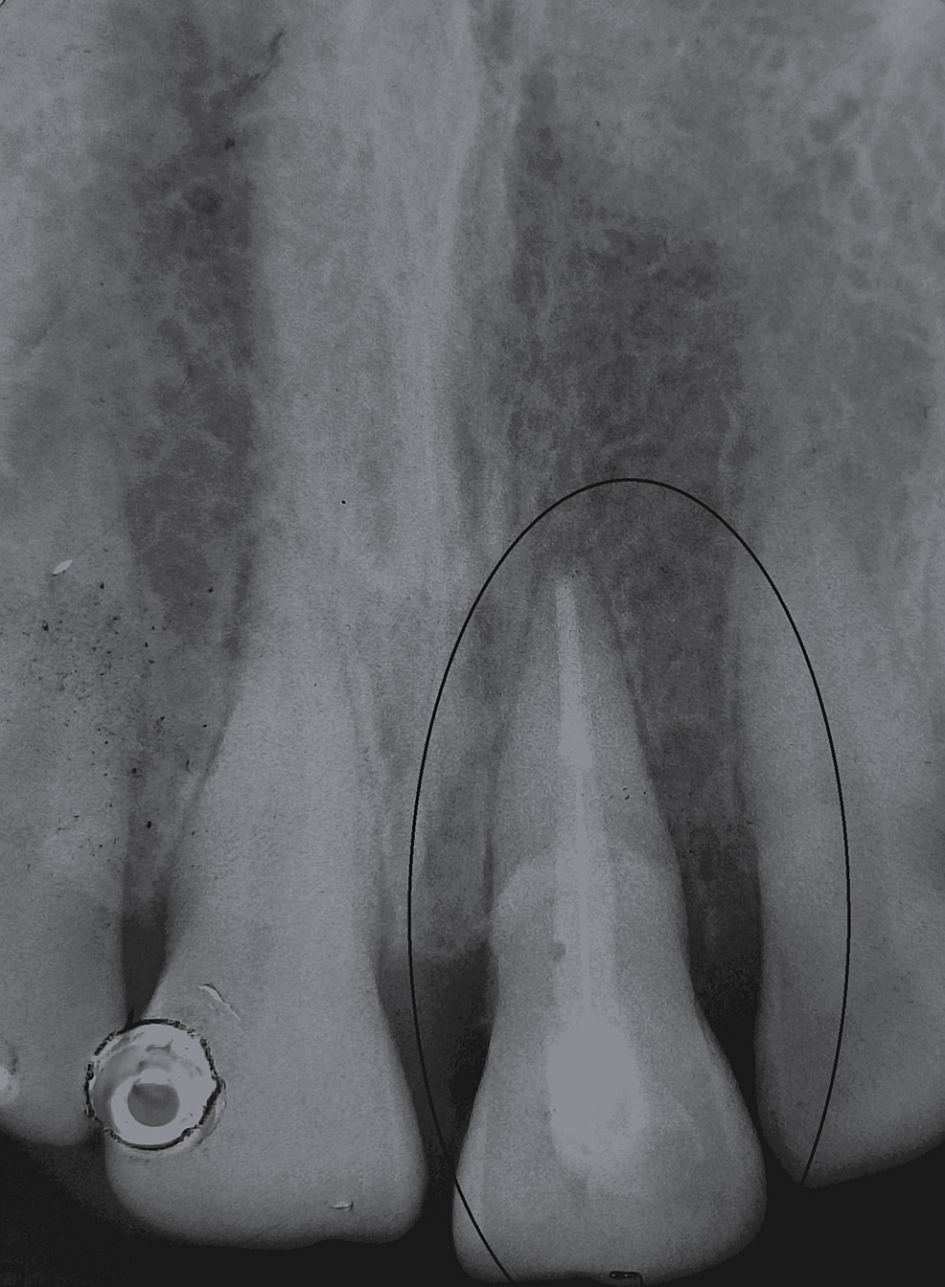
Radiographic evaluation of tooth 21 after 18 months of fragment reattachment

On the 18-month follow-up with tooth 21 reattached, the tooth fragment showed a proper adaptation as well as good periodontal health with intact lamina dura and no evidence of root resorption (Figures 10, 11). However, a good prognosis is only possible when patients cooperate and understand the limitations of the treatment.

## Discussion

In a young individual, trauma to the anterior teeth is a tragic experience requiring immediate attention, as it can damage the patient's dentition as well as affect the patient's psychology. An immediate restorative technique for a fractured anterior tooth is to reattach the fractured tooth segment. The procedure restores the morphological, esthetic, and functional aspects of the dentition.

For reattachment to be successful, a number of factors must be considered, including the site of the fracture, the size of the fractured remnant, pulpal involvement, root maturity, periodontal status, biological width invasion, occlusion, and the material used for reattachment. The longer the fragment remains dehydrated, the tooth strength will be impaired. By rehydrating the fragment of the tooth, tooth resistance can be improved. Since dentin dehydration causes the collapse of collagen fibers, this results in inadequate penetration of resin monomers, which will lead to poor adhesion between the dentin and composite. The patient presented to the department immediately post-trauma with an intact fractured segment. Thus, the fractured segment was much less likely to become dehydrated.

The direction of the fracture line is a key consideration in restorability, as it directly influences the tooth's prognosis. Accordingly, in the above case report, the fractured fragment was in good condition and had a proper fit over the radicular portion. Therefore, the reattachment technique with fiber post reinforcement was most appropriate. In the past, cast metal core and posts were used for fracture reattachment. Some of the newer varieties of nonmetallic posts are made from ceramic or fiber-reinforced materials, such as quartz, carbon, or glass combined with an epoxy matrix. The advantages of tooth-colored fiber posts are numerous [9]. These are more esthetic, bond to the tooth with a similar modulus of elasticity as that of dentin, and have a low fracture rate. Recent advances in adhesive techniques and materials have made it possible to fabricate a monoblock, a multilayered structure without weak interlayer interfaces, using glass fiber posts with a composite core. By using this concept, this system reinforces the tooth structure. Therefore, the final endodontic continuum monoblock has the same integrity as the original healthy tooth [10]. Additionally, fiber posts allow stress to be distributed to the remaining radicular dentin. In addition to strengthening the tooth, luting the fiber posts with resin cement increases the bond strength of the fractured segment. Furthermore, it reduces the inclusion of air voids, provides predictable results, and is easy to use. Light-cured luting resin cement can result in incomplete polymerization in apical areas. Therefore, dual-curing systems are more suitable since they allow for polymerization of even those areas that otherwise would have been left uncured because of the insufficient light reaching deeper areas. A resin-based sealer is typically used for obturating the teeth that will be restored by glass fiber posts since the setting of resin cement may be inhibited by eugenol-based sealers [10].

This case involved reattaching the tooth with no preparation technique. As per Shirani et al., reattachment of the fragment with no preparation technique and adequate rehydration resulted in higher bond strength [11,12]. Studies by Worthington et al. and Davari and Sadeghi found that reattached teeth with added preparations such as bevels, over contour, and the internal grooves do not provide better bond strength than that of no preparation technique [13]. As an intermediate restorative material, resin cement was used for reattachment since its dual-cure mechanism provides better bond strength.

Reattaching teeth fragments, especially in young patients, has some advantages over conventional composite and prosthetic restorations; however, fractures that extend subgingivally are particularly difficult to treat and have a low healing rate. The biological width is the sum of the epithelial and connective tissue attachment lengths. It has been stated that when the fracture invades the biologic width, flap surgery should be performed with minimal osteotomy and osteoplasty. However, Ramfjord reported that in situations with minimal biologic width invasion, the organism is able to restore biologic width by itself, provided the dental plaque is properly controlled [14]. In the above case, considering that the biologic width invasion was minimal and supraosseous, the fracture line was exposed through periodontal flap reflection. The success rate of the reattached teeth is also determined by the fit, contour, and surface finish of the subgingival restoration. The patient was reviewed after 18 months and clinical and radiographic outcomes were satisfactory with normal contour and appearance.

The purpose of this case report was to demonstrate the multidisciplinary approach to treating dental trauma and its possible sequelae, especially in cases of unusual tooth fracture.

## Conclusions

The treatment strategy for complicated crown-root fracture is complicated because of the sub-gingival position of the fracture margin. Several factors should be considered when choosing a technique or material for fragment reattachment. Occlusion relation affects the treatment success and outcome in cases of complicated crown-root fracture. An optimal prognosis is obtained when adjacent teeth and opposing teeth are in proper alignment. Following 18 months of follow-up, a successful outcome was obtained in this case report. The reattachment procedure is relatively simple and the tooth can be immediately restored after an injury. Especially in younger patients with anterior tooth fracture, this technique should be considered when the fractured fragment is available. It is important to follow cases of complicated root-crown fracture regularly. Any complication may be identified early and treated. However, patients must be informed of the possibility of interim treatment.

## References

[1] Holan G, Shmueli Y (2003). Knowledge of physicians in hospital emergency rooms in Israel on their role in cases of avulsion of permanent incisors. Int J Paediatr Dent.

[2] Krishna A, Malur MH, Swapna DV, Benjamin S, Deepak CA (2012). Traumatic dental injury—an enigma for adolescents: a series of case reports. Case Rep Dent.

[3] Andreasen JO, Ravn JJ (1972). Epidemiology of traumatic dental injuries to primary and permanent teeth in a Danish population sample. Int J Oral Surg.

[4] Andreasen JO (1970). Etiology and pathogenesis of traumatic dental injuries. A clinical study of 1,298 cases. Scand J Dent Res.

[5] Buonocore MG, Davila J (1973). Restoration of fractured anterior teeth with ultraviolet-light-polymerized bonding materials: a new technique. J Am Dent Assoc.

[6] Rajput A, Talwar S, Ataide I, Verma M, Wadhawan N (2011). Complicated crown-root fracture treated using reattachment procedure: a single visit technique. Case Rep Dent.

[7] Arhun N, Ungor M (2007). Re-attachment of a fractured tooth: a case report. Dent Traumatol.

[8] Garcia FCP, Poubel DLN, Almeida JCF, Toledo IP, Poi WR, Guerra ENS, Rezende LVML (2018). Tooth fragment reattachment techniques—a systematic review. Dent Traumatol.

[9] Tay FR, Pashley DH (2007). Monoblocks in root canals: a hypothetical or a tangible goal. J Endod.

[10] Thapak G, Arya A, Arora A (2019). Fractured tooth reattachment: a series of two case reports. Endodontology.

[11] Shirani F, Malekipour MR, Sakhaei Manesh V, Aghaei F (2012). Hydration and dehydration periods of crown fragments prior to reattachment. Oper Dent.

[12] Shirani F, Malekipour MR, Tahririan D, Sakhaei Manesh V (2011). Effect of storage environment on the bond strength of reattachment of crown fragments to fractured teeth. J Conserv Dent.

[13] Khandelwal P, Srinivasan S, Arul B, Natanasabapathy V (2021). Fragment reattachment after complicated crown-root fractures of anterior teeth: a systematic review. Dent Traumatol.

[14] Ramfjord SP (1988). Periodontal considerations of operative dentistry. Oper Dent.

